# Different antiviral effects of IFNα subtypes in a mouse model of HBV infection

**DOI:** 10.1038/s41598-017-00469-1

**Published:** 2017-03-23

**Authors:** Jingjiao Song, Sheng Li, Yun Zhou, Jia Liu, Sandra Francois, Mengji Lu, Dongliang Yang, Ulf Dittmer, Kathrin Sutter

**Affiliations:** 1 Experimental Medicine Center, Tongji Hospital, Tongji Medical College, Huazhong University of Science and Technology, Wuhan, P.R. China; 2 Department of Infectious Diseases, Union Hospital of Tongji Medical College, Huazhong University of Science and Technology, Wuhan, P.R. China; 3 Institute of Virology, University Hospital of Essen, University of Duisburg-Essen, Essen, Germany

## Abstract

Interferon alpha (IFNα) is commonly used for the treatment of chronic hepatitis B (CHB) patients. There are 13 different IFNα subtypes in humans, but only the subtype IFNα2 is used for clinical treatment. The antiviral activities of all other IFNα subtypes against HBV have not been studied. To obtain basic knowledge about the direct antiviral as well as the immunomodulatory effects of IFNα subtypes, we used the HBV hydrodynamic injection (HI) mouse model. Application of most IFNα subtype proteins inhibited HBV replication *in vivo*, with IFNα4 and IFNα5 being the most effective subtypes. Decreased viral loads after therapeutic application of IFNα4 and IFNα5 correlated with expanded effector cell populations of NK cells and T cells in both liver and spleen. Hydrodynamic injection of plasmids encoding for the effective IFNα subtypes (pIFNα) was even more potent against HBV than injecting IFNα proteins. The combination of pIFNα4 and pIFNα5 showed a synergistic antiviral effect on HBV replication, with a strong increase in NK cell and T cell activity. The results demonstrate distinct anti-HBV effects of different IFNα subtypes against HBV in the mouse model, which may be relevant for new therapeutic approaches.

## Introduction

Hepatitis B virus (HBV) infection is one of the major threats to public health worldwide and more than 240 million people are currently infected. Approximately 25% of these individuals develop HBV-associated diseases, including liver failure, cirrhosis and hepatocellular carcinoma (HCC)^1^.

Host immune responses are very important to determine the outcome of HBV infection, with T cells playing a fundamental role in HBV clearance and pathogenesis. Cytotoxic CD8^+^ T cells (CTL) can control viral infection by killing virus-infected cells through various effector molecules (Granzymes, TRAIL, FasL). The numbers of CTL in the liver are very limited, thus non-cytopathic effector functions of CD4^+^ and CD8^+^ T cells, like the production of antiviral cytokines (IFN-γ, TNFα), are indispensable to control HBV infection^2, 3^. During acute HBV infection virus-specific CD8^+^ T cells are required for the control and elimination of HBV infection and the strength of the HBV-specific CD8^+^ T cell response correlates with viral clearance^4^. Previous studies in HBV-infected chimpanzees also reported, that the depletion of CD8^+^ T cells during acute infection led to sustained high viral titers^5^, emphasizing their importance in viral control. During chronic HBV infection, HBV-specific CD8^+^ T cells are only barely detectable in patients with high viremia^6^. These virus-specific CD8^+^ T cells are either deleted during infection^7^, insufficiently primed by antigen-presenting cells^8, 9^ or functionally exhausted characterized by the expression of inhibitory molecules like PD-1, CTLA4 or Tim-3^10–12^.

NK cells represent the main effector population of the innate immune system against viruses^13^, and these lymphoid effector cells are abundant in liver tissue^14^. Contradictory data exist about the exact role of NK cells in acute and chronic HBV infection. During acute HBV infection liver NK cells were characterized by increased cytolytic activity and IFNγ production in comparison to NK cells in chronic HBV infection^15, 16^. In contrast, others reported an impaired function of NK cells in patients with acute HBV infection compared to healthy individuals due to increased IL-10 serum levels^17^. In chronic HBV patients (CHB) altered expression of ligands for activating and inhibitory NK cell receptors was shown to result in impaired NK cell effector functions^18^. High levels of IL-10 in CHB patients can further suppress NK cell cytotoxicity^19^.

As effective NK cell and CD8^+^ T cell responses are required for controlling and resolving HBV infection, the development of immunotherapies, which directly improve these host immune responses are of special interest. The current treatment of CHB patients includes IFNα and nucleos(t)ide analogues. IFNα is able to induce numerous IFN-stimulated genes (ISGs) which exhibit antiviral as well as immunomodulatory activity. In addition, IFNα stimulates NK cells, macrophages and dendritic cells to regulate host innate immune responses^20^, but it also modulates T cell and B cell responses. Several previous studies suggested that IFNα therapy has long-term beneficial effects in terms of viral clearance, prevention of HCC and prolonged survival in patients with CHB^21, 22^. However, the therapeutic success of the current IFNα treatment of CHB patients is limited. Only up to 30% of the CHB patients show a sustained response to IFNα therapy, which restricts the clinical benefit of IFNα^23^. Up to now, only one subtype (IFNα2) out of 12 different human IFNα subtypes is used in clinical treatments. Although all IFNα subtypes bind the same receptor (IFNAR1/2) they all differ in their biological activities. The mechanisms of the varying biological effects of these highly conserved proteins (75–99% amino acid sequence identity)^24^ are not completely understood. One possible explanation is that the binding affinities to the receptor subunits differ considerably between the subtypes^25^. IFNα subtypes are also able to activate various downstream signaling pathways^26^, which might result in the induction of distinct expression patterns of ISGs^27–29^. Therefore, the outcome of IFNα treatment strongly depends on the individual subtype. The antiviral effects of other IFNα subtypes against HBV infection were not tested so far.

A strong host immune response is needed to control and resolve HBV infection. However, to develop new immunotherapies against HBV which directly target host NK or T cells, detailed analysis of the immunomodulatory effects of such a therapy *in vivo* is required. As *in vitro* cell culture systems are limited for the investigation of complex host immune responses, other *in vivo* model systems should be used to clarify immune responses against HBV. Mice cannot be infected with HBV, whereas using the well-established HBV hydrodynamic injection (HI) mouse model, we are able to stably transfect mouse hepatocytes with an HBV expression plasmid. This leads to the induction of a host immune response against HBV in the liver and thus mimicking HBV infection *in vivo*. In a previous study, we already showed that one single IFNα subtype (IFNα4) had different antiviral effects against two HBV isolates using the HI mouse model^30^. Thus, further investigations to determine the antiviral and in particular the immunomodulatory activities of various IFNα subtypes against HBV are of great interest. In this study, we primarily investigated the antiviral efficacies of different murine IFNα subtypes against HBV *in vivo*. We further applied the most potent IFNα subtypes as recombinant proteins during infection as well as plasmids encoding for the effective subtypes. Plasmid application resulted in long-term expression of IFNα in the liver. Both experimental setups led to reduced HBV replication and antigen expression in the liver and IFNα4 and IFNα5 were the most effective subtypes which were able to suppress HBV replication. Both IFNα treatment regimens significantly improved NK and T cell effector functions correlating with accelerated viral clearance in the HBV HI mouse model.

## Results

### Inhibition of HBV after treatment with recombinant IFNα4 and IFNα5 proteins

**Figure 1:**
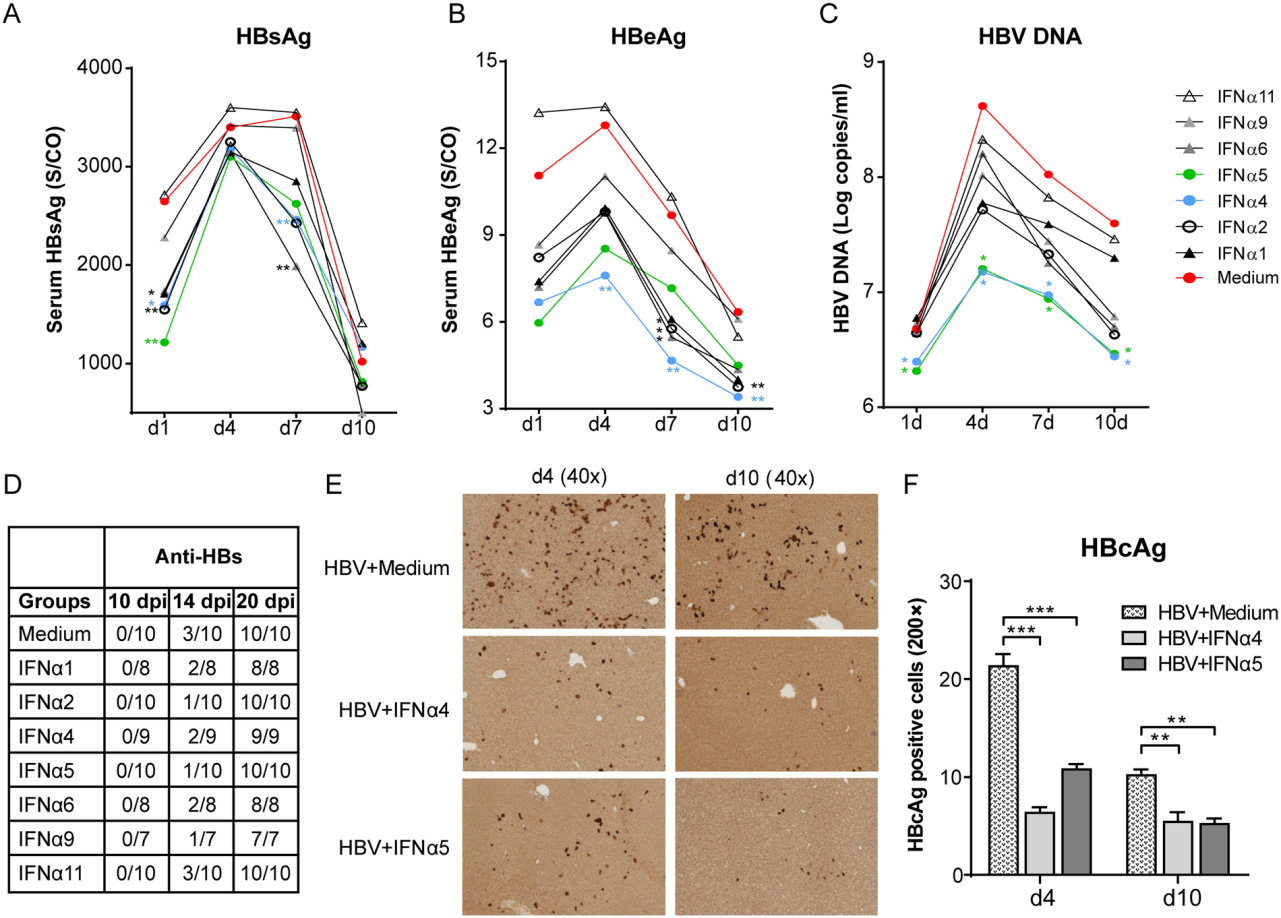
Kinetics of HBV replication in mice treated with different IFNα subtype proteins. Mice received HI with 10 µg of pAAV-HBV1.2 plasmids. Mice were treated i.p. with 8000 units of recombinant IFNα1, α2, α4, α5, α6, α9 or α11 proteins starting from days −1 to 9 post HI. Mouse sera were collected at the indicated time points. (**A**) HBsAg, (**B**) HBeAg, (**C**) qPCR detection of HBV DNA levels and (**D**) anti-HBs in the sera of mice after HI. At least six mice per group were analyzed. At days 4 and 10 post HI, mice were sacrificed and livers were analyzed. Immunohistochemical stainings using anti-HBc antibodies (**E**) were performed in liver sections of mice and frequencies of HBcAg positive cells are shown (**F**). At least four mice per group were analyzed. The data were analyzed by One-way ANOVA. Statistically significant differences between the IFN-treated groups and the untreated control group are indicated by * for p < 0.05, ** for p < 0.01 and *** for p < 0.001.

IFNα2 is clinically used to treat CHB patients, however only up to 30% of the patients develop a sustained viralogical response. Other immunotherapies are needed to further improve anti-HBV treatment. In a previous study we have already analyzed the antiviral effect of one murine IFNα subtype (IFNα4) against different HBV stains *in vivo* using the HI mouse model. Understanding the biology of the pleiotropic IFNα subtypes might be useful to develop future immunotherapies against HBV. In the current work we aimed to investigate the antiviral effects of various mouse IFNα subtypes against HBV *in vivo* as well as their stimulatory effect on host innate and adaptive immune responses against HBV. For that purpose we hydrodynamically injected a plasmid encoding for HBV into Balb/c mice and treated them intraperitoneally (i.p.) with different IFNα subtype proteins daily starting one day prior HI. At days 1, 4, 7 and 10 we collected serum samples of all mice and analyzed HBsAg (Fig. 1A), HBcAb (data not shown) and HBeAg (Fig. 1B) concentrations in the serum as well as HBV DNA (Fig. 1C). Figure 1A shows the kinetics of HBsAg concentration in the serum with peak HBsAg levels at 4–7 days post HI, which rapidly declined in all mice at day 10. At the day of maximum HBsAg level, all IFNα subtypes except IFNα11 were able to decrease HBsAg levels compared to control mice which received daily infections of medium (red dot). Similar results were detected for HBeAg (Fig. 1B) and HBV DNA (Fig. 1C), nonetheless two IFNα subtypes, IFNα4 (blue) and IFNα5 (green), were the most potent antiviral subtypes against HBV *in vivo*. In contrast, no induction of anti-HBc antibodies was detectable in all investigated groups at days 4 and 10 post HI (data not shown). We also analyzed antibody responses against HBsAg at 10, 14 and 20 days post HI (Fig. 1D). At 10 days no antibodies were detectable in any of the groups, however at later time points all mice were positive for anti-HBs antibodies and no significant differences between the groups were observed. The following experiments were performed with 2 IFNα subtypes (IFNα4 and α5) and further parameters of infection in the liver were analyzed. At days 4 and 10 post HI immunohistochemical stainings of liver sections for HBcAg expression were performed in IFN-treated and control mice. As depicted in Fig. 1E and 1F, daily application of IFNα4 or IFNα5 protein significantly decreased the numbers of HBcAg positive cells in the liver compared to untreated control mice (HBV + Medium). These results highlight the high antiviral potency of IFNα4 and IFNα5 against HBV *in vivo*.

### IFNα4 and IFNα5 enhanced the expression of ISG15, OAS and PKR in the liver

**Figure 2:**
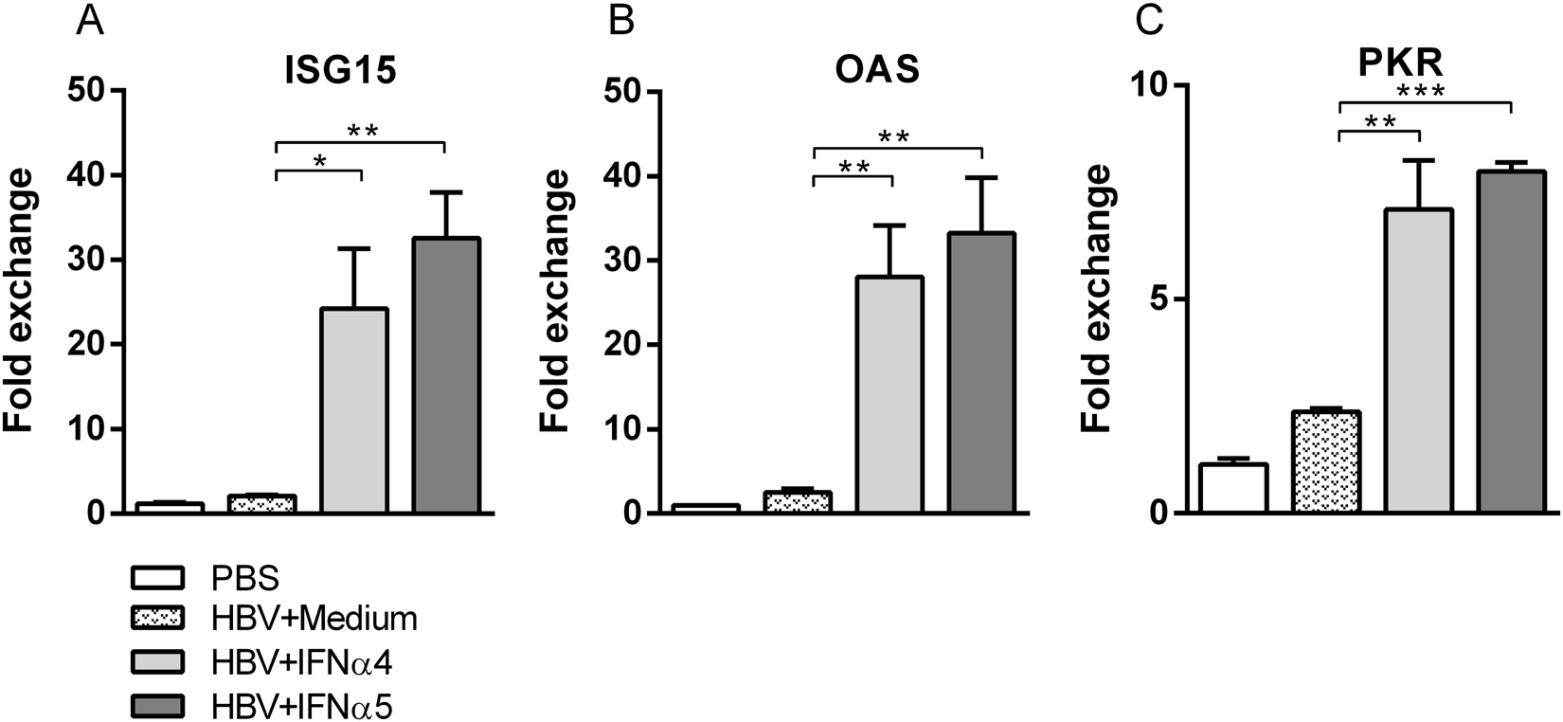
Induction of ISG15, OAS and PKR expression post treatment with recombinant IFNα4 or IFNα5. Mice received HI with 10 µg of pAAV-HBV1.2 plasmids. Mice were treated i.p. with 8000 units of recombinant IFNα4 or α5 protein starting from days −1 to 3 post HI. Control mice received either HI with PBS (PBS) or were treated i.p. with media (HBV + Medium). Total RNA was extracted from liver tissue at day 4 post HI and the mRNA expression levels of (**A**) ISG15, (**B**) OAS and (**C**) PKR were determined by qRT-PCR. The β-actin mRNA expression was quantified for normalization. Each sample was run in duplicate and at least four mice per group were analyzed. Differences between the groups were analyzed by using the One-way ANOVA. Statistically significant differences between the IFN-treated groups and the untreated control group are indicated by * for p < 0.05, ** for p < 0.01 and *** for p < 0.001.

To find a mechanistic correlate for the anti-HBV activity of IFNα subtypes 4 and 5 *in vivo*, we analyzed the expression of the IFN-stimulated genes (ISG) ISG15, 2′-5′-oligoadenylate synthase (OAS) and Proteinkinase R (PKR), which can directly block viral replication and protein synthesis. In comparison to uninfected control mice (PBS), HI of the HBV plasmid did not induce the expression of the three investigated ISGs. In contrast, treatment with IFNα4 or IFNα5 significantly increased the mRNA expression of all three ISGs (Fig. 2), with slightly higher induction upon stimulation with IFNα5. However, all previously tested IFNα subtypes (IFNα1, α2, α6, α9 and α11) were also able to increase the ISG expression comparable with IFNα4 and IFNα5 (Supp. Fig. 1), indicating that the induction of antiviral ISGs alone is not sufficient to completely control HBV replication *in vivo*.

### Immunomodulatory effects of IFNα subtypes on NK cell responses

**Figure 3:**
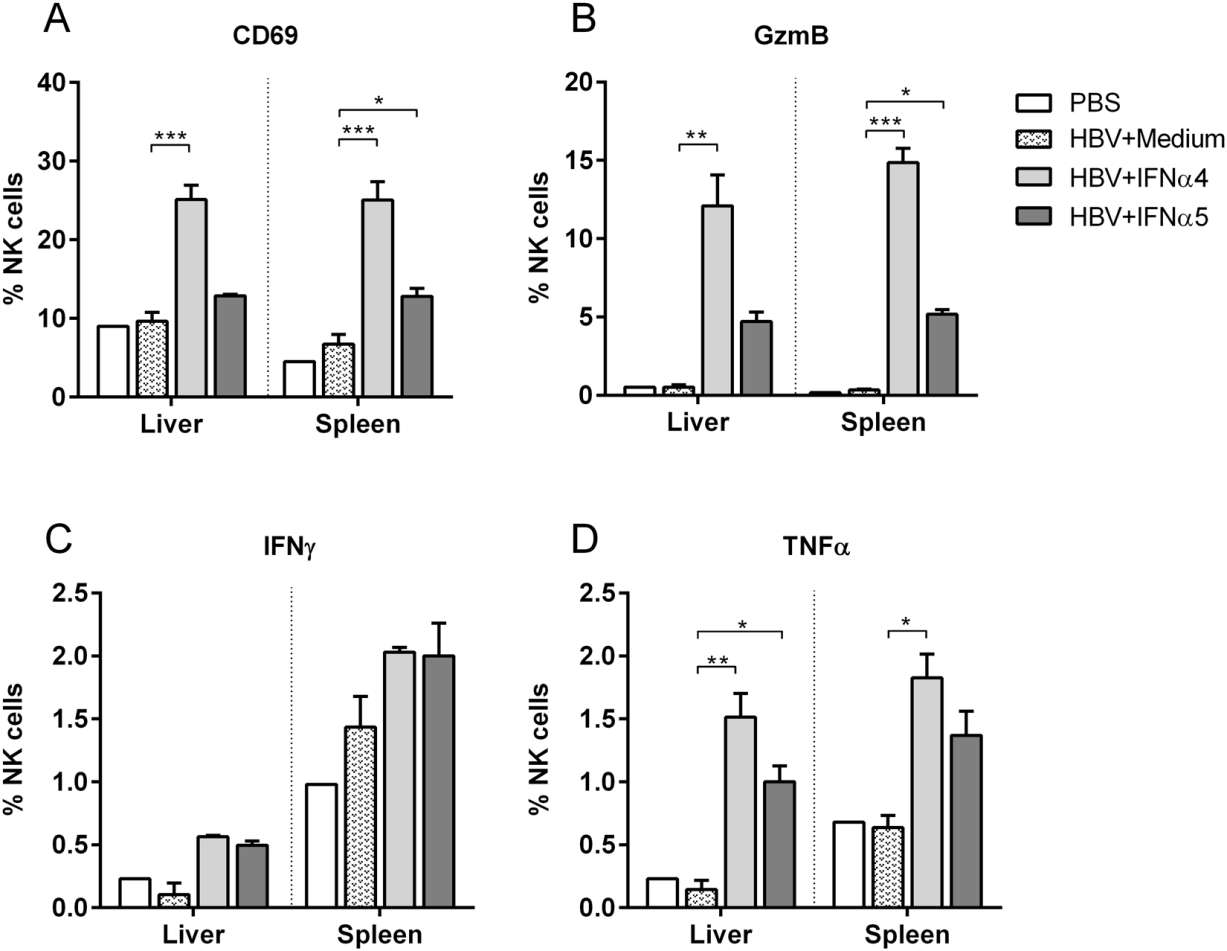
Analysis of NK cells from IFNα4 or IFNα5-treated mice. Mice received HI with 10 µg of pAAV-HBV1.2 plasmids. Mice were treated i.p. with 8000 units of recombinant IFNα4 or α5 protein starting from days −1 to 3 post HI. Control mice received either HI with PBS (PBS) or were treated i.p. with media (HBV + Medium). At day 4 post HI mice were sacrificed and nucleated cells from liver and spleen were analyzed by flow cytometry for expression of NK cell-specific surface markers and intracellular cytokines. (**A**) The percentage of activated CD69^+^ NK cells, (**B**) GzmB, (**C**) IFNγ and (**D**) TNFα-producing NK cells were shown. At least four mice per group were analyzed. Differences between the groups were analyzed by using the One-way ANOVA. Statistically significant differences between the IFN-treated groups and the untreated control group are indicated by * for p < 0.05, ** for p < 0.01 and *** for p < 0.001.

As the beneficial outcome of an IFNα therapy in patients depends on direct antiviral and immunomodulatory activities, we determined host immune responses under IFNα subtype treatment. Firstly, we elucidated the impact of IFNα therapy on NK cell responses, which were shown to be important in controlling HBV infection. Figure 3A shows that HI of the HBV plasmid alone did not lead to the activation of NK cells in the liver or spleen as measured by the expression of the early activation marker CD69. Only injections of IFNα4 protein significantly increased the frequencies of activated NK cells in the liver. In contrast, treatment with IFNα5 did not influence NK cell activation. Next, we analyzed the effector phenotype of NK cells during HBV infection and IFNα subtype treatment *in vivo*. No significant difference in the percentages of NK cells expressing IFNγ was detectable post IFN treatment (Fig. 3C), in contrast to the induction of NK cells expressing granzyme B and TNFα. IFNα4 significantly increased the frequencies of cells producing the cytolytic protease granzyme B (Fig. 3B) and the cytokine TNFα (Fig. 3D) compared to untreated control mice (HBV + Medium). IFNα5 only elevated the frequencies of TNFα-expressing NK cells (Fig. 3D), but no effect on granzyme B expression was observed (Fig. 3B). Similar effects on NK cell responses were also detected in the spleen after IFNα4 and α5 treatment. These results further imply that, apart from its direct antiviral effect, IFNα4 or α5 stimulate anti-viral effector functions of NK cells during HBV infection likely contributing to the control of viral replication.

### IFNα4 strongly augmented T cell responses against HBV *in vivo*

**Figure 4:**
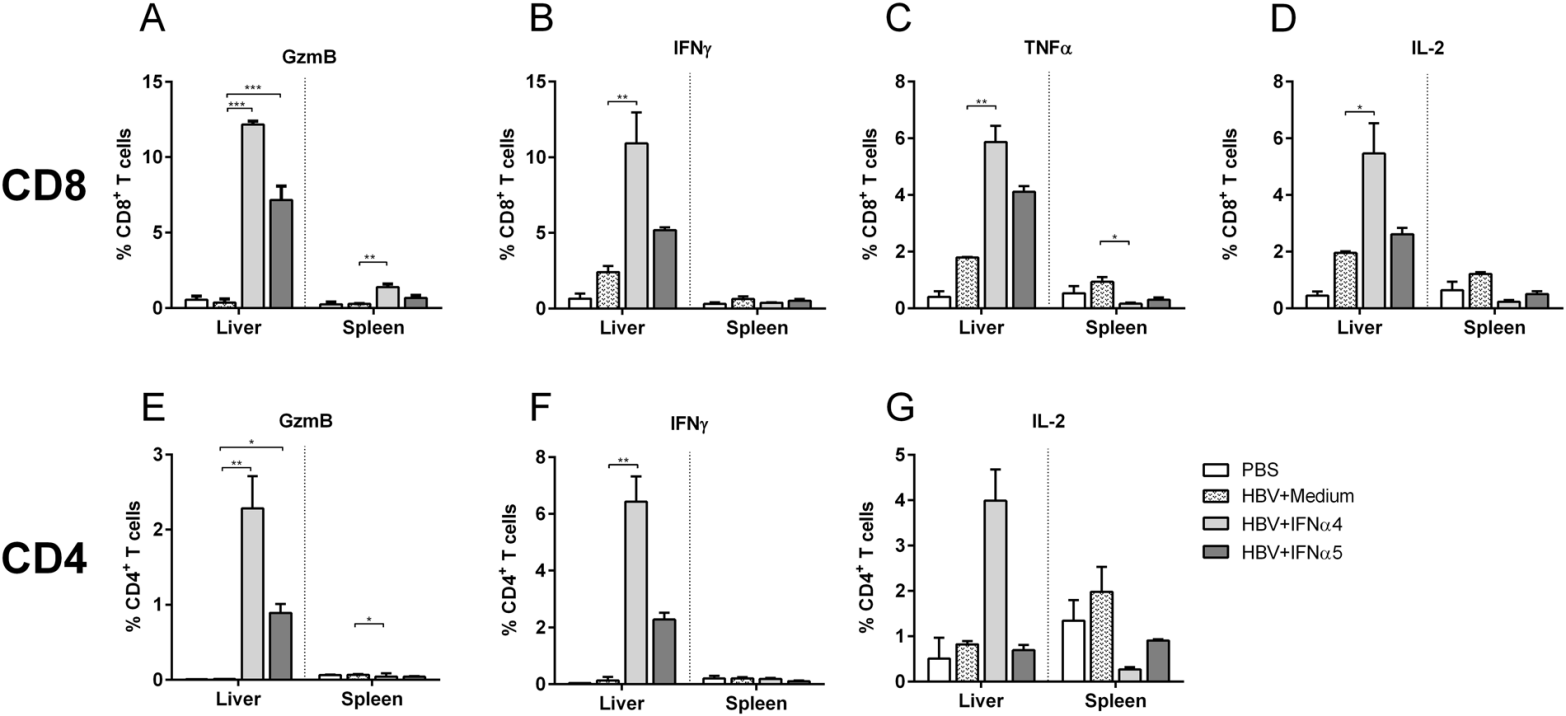
Analysis of CD8^+^ and CD4^+^ T cells from IFNα4 or IFNα5-treated mice. Mice received HI with 10 µg of pAAV-HBV1.2 plasmids. Mice were treated i.p. with 8000 units of recombinant IFNα4 or α5 protein starting from days −1 to 9 post HI. Control mice received either HI with PBS (PBS) or were treated i.p. with media (HBV + Medium). At day 10 post HI mice were sacrificed and nucleated cells from liver and spleen were analyzed by flow cytometry for expression of T cell-specific surface markers and intracellular cytokines. Frequencies of GzmB (**A**), IFNγ (**B**), TNFα (**C**) and IL-2- producing CD8^+^ T cells (**D**), GzmB (**E**), IFNγ (**F**) and IL-2-producing CD4^+^ T cells (**G**) were shown. At least four mice per group were analyzed. Differences between the groups were analyzed by using the One-way ANOVA. Statistically significant differences between the IFN-treated groups and the untreated control group are indicated by * for p < 0.05, ** for p < 0.01 and *** for p < 0.001.

Other immune cells, which are essential to control and resolve HBV infection, are CD8^+^ and CD4^+^ T cells. In our experimental setup we examined if T cell responses were affected by IFNα protein treatment *in vivo*. Therefore we hydrodynamically injected the HBV plasmid into Balb/c mice and treated them with recombinant IFNα4 or α5 protein from day −1 to day 9 post HI. At day 10 mice were sacrificed and T cell responses in liver and spleen were extensively studied (Fig. 4). As presented in Fig. 4A–D, CD8^+^ T cell responses were barely detectable in both organs of HBV-infected untreated control mice (HBV + Medium). In contrast treatment with IFNα strongly increased frequencies of CD8^+^ T cells in the liver, but responses in the spleen were only weak (Fig. 4A). In addition, these expanded CD8^+^ T cells expressed granzyme B (Fig. 4A), IFNγ (Fig. 4B), TNFα (Fig. 4C) and IL-2 (Fig. 4D). In particular, IFNα4 potently improved CD8^+^ T cell responses. Up to 30 times higher frequencies of granzyme B expressing CD8^+^ T cells were measured in the liver of IFNα4 treated mice compared to control mice (HBV + Medium). Administration of IFNα5 resulted in an 18 fold increase in the frequencies of granzyme B expressing CD8^+^ T cells, whereas the percentages of cytokine expressing cells was not significantly augmented by IFNα5. In contrast to NK cell responses stimulated by IFNα4 or IFNα5 treatment (Fig. 3), CD8^+^ T cell responses were only locally induced in the liver by recombinant IFNα protein injections, but not in the spleen. We also analyzed CD4^+^ T cell responses in the liver of HBV-infected mice. Injection of HBV plasmid alone did not induce any detectable CD4^+^ T cell response (Fig. 4E–G). The daily application of IFNα5 only slightly increased the frequencies of granzyme B (Fig. 4E) or cytokine expressing CD4^+^ T cells (Fig. 4F,G), whereas treatment with IFNα4 significantly increased the frequencies of those cells in the liver. Especially the percentage of IFNγ-producing CD4^+^ T cells was 50 fold higher compared to control mice (HBV + Medium) after IFNα4 therapy. Again, no effect of the treatment was found in the spleen HBV HI mice. These data demonstrate that especially immunotherapy with recombinant IFNα4 protein significantly improves CD4^+^ and CD8^+^ T cell responses in HBV HI mouse model.

### Anti-HBV effects were enhanced by application of the pIFNα4 and pIFNα5

**Figure 5:**
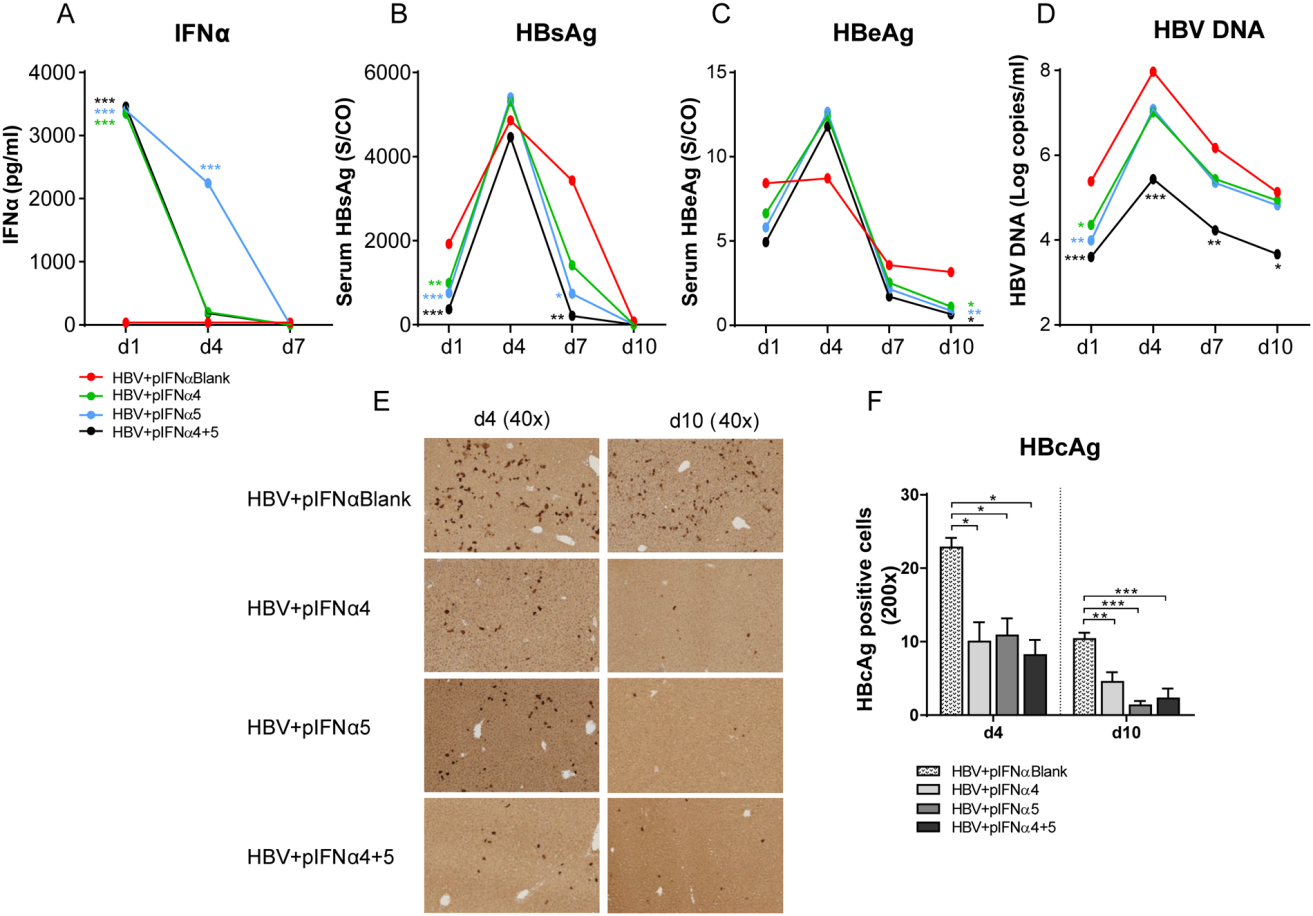
Kinetics of HBV replication in mice treated with plasmids encoding for different IFNα subtypes. Mice received HI with 10 µg of pAAV-HBV1.2 plasmids in combination with 20 µg of plasmids encoding for IFNα subtypes (pIFNα4, pIFNα5, pIFNα4 + 5 or pIFNαBlank (empty vector)). Mouse sera were collected at the indicated time points. (**A**) IFNα protein levels, (**B**) HBsAg, (**C**) HBeAg and (**D**) qPCR detection of HBV DNA levels in the sera of mice after HI. At days 4 and 10 post HI, mice were sacrificed and livers were analyzed. Immunohistochemical stainings using anti-HBc antibodies (**E**) were performed and frequencies of HBcAg positive cells are shown (**F**). At least six mice per group were analyzed. The data were analyzed by One-way ANOVA. Statistically significant differences between the IFN-treated groups and the untreated control group are indicated by * for p < 0.05 ** for p < 0.01 and *** for p < 0.001.

To further boost the IFNα-mediated anti-HBV effects and host immune response, we performed additional experiments where we used plasmids encoding for the IFNα subtypes 4 and 5, which should result in long-lasting endogenous IFNα expression in the liver of HBV HI mice. Here, we hydrodynamically injected these plasmids together with the HBV-encoding plasmid into mice. In our previous experiments we showed that injection of recombinant IFNα5 protein induced the highest ISG expression levels in the liver (Fig. 2), whereas application of recombinant IFNα4 protein specifically improved host NK and T cell responses (Figs 3 and 4). Thus we used here another experimental group of mice which received a combination of both plasmids encoding for IFNα4 and α5 to figure out if this could further increase the therapeutic effect of IFN during HBV *in vivo*. As control an empty plasmid (pIFNαBlank) together with the HBV encoding plasmid were hydrodynamically injected into the mice. At days 1, 4 and 7 we collected serum samples of all mice and analyzed the concentration of IFNα by ELISA (Fig. 5A). HBV itself did not induce IFNα (HBV + pIFNαBlank), whereas all mice receiving plasmids encoding for IFNα subtypes had detectable levels of IFNα at days 1 and 4 post application. The concentrations of HBsAg (Fig. 5B) and HBeAg (Fig. 5C) were reduced upon pIFNα treatment. At peak viremia (d4) injection of either pIFNα4 or pIFNα5 significantly reduced HBV DNA (1.03 × 10^7^ and 1.23 × 10^7^ HBV DNA copies per ml serum, respectively) compared to control mice (9.36 × 10^7^ HBV DNA copies per ml serum). However, mice which got both IFNα expressing plasmids had a massive reduction in HBV DNA (2.7 × 10^5^ HBV DNA copies per ml serum at d4) suggesting that the therapy with both IFNα encoding plasmids exert the highest antiviral potency against HBV *in vivo* (Fig. 5D). We did not detect any anti-HBc antibodies in all investigated groups at days 4 and 10 post HI (data not shown).

At days 4 and 10 post HI immunohistochemical stainings of liver sections were done for HBcAg expression in pIFNα-treated and control mice. Compared to untreated control mice (HBV + pIFNαBlank) application of pIFNα4 or pIFNα5 or the combination of both significantly decreased the numbers of HBcAg positive cells to a similar extent (Fig. 5E,F). All three treatment regimen using different IFNα expression plasmids were highly effective in suppressing HBV replication *in vivo*.

### pIFNα induced higher mRNA expression of ISG15, OAS and PKR in the liver

**Figure 6:**
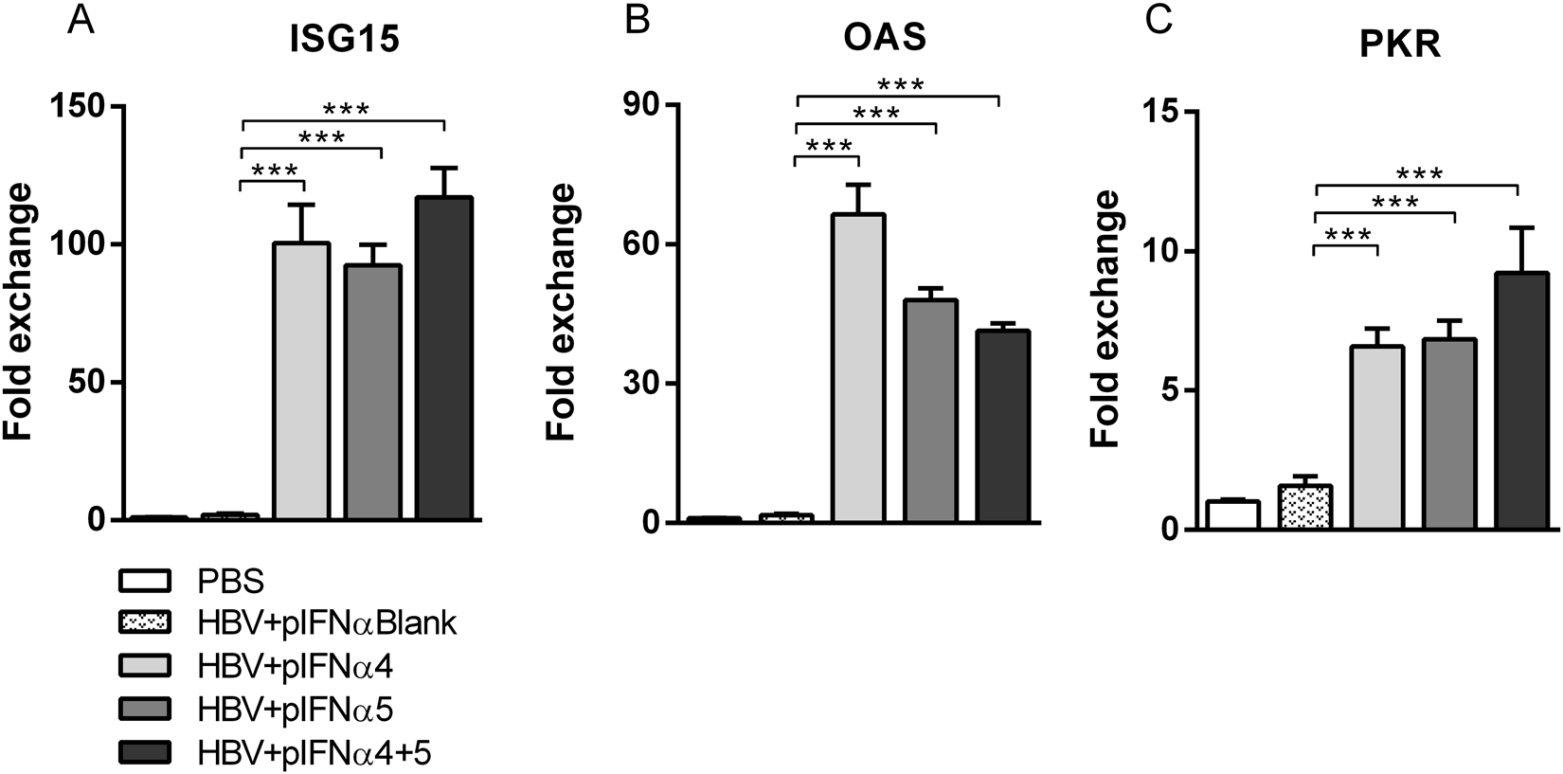
Induction of ISG15, OAS and PKR expression post treatment with plasmids encoding for different IFNα subtypes. Mice received HI with 10 µg of pAAV-HBV1.2 plasmids in combination with 20 µg of plasmids encoding for IFNα subtypes (pIFNα4, pIFNα5, pIFNα4 + 5 or pIFNαBlank (empty vector)). Total RNA was extracted from liver tissue at day 4 post HI and the mRNA expression levels of (**A**) ISG15, (**B**) OAS and (**C**) PKR were determined by qRT-PCR. The β-actin mRNA expression was quantified for normalization. Each sample was run in duplicate and at least four mice per group were analyzed. Differences between the IFN-treated groups and the untreated control group were analyzed by using the One-way ANOVA. Statistically significant differences between the groups are indicated by *** for p < 0.001.

As hydrodynamic infection of plasmids encoding for different IFNα subtypes significantly reduced HBV replication, we were interested in the intrahepatic expression of ISGs during HBV infection and pIFNα treatment. Application of pIFNα4, pIFNα5 or the combination of both plasmids significantly increased the mRNA expression of all three ISGs (Fig. 6) to similar extent. In comparison to the therapy with the recombinant IFNα proteins (Fig. 2), the endogenous expression of IFNα in the liver strongly enhanced the mRNA expression of all 3 ISGs. ISG15 was up to 60 fold increased upon treatment with both IFNα-expressing plasmids, whereas IFNα5 protein treatment increased the ISG15 mRNA expression only up to 16 fold compared to untreated control mice (Fig. 2A). These results highlight that endogenous expression of IFNα4 or α5 in the liver further increased the induction of ISGs with potential anti-HBV activity *in vivo*.

### Immunomodulatory functions of pIFNα on NK cells

**Figure 7:**
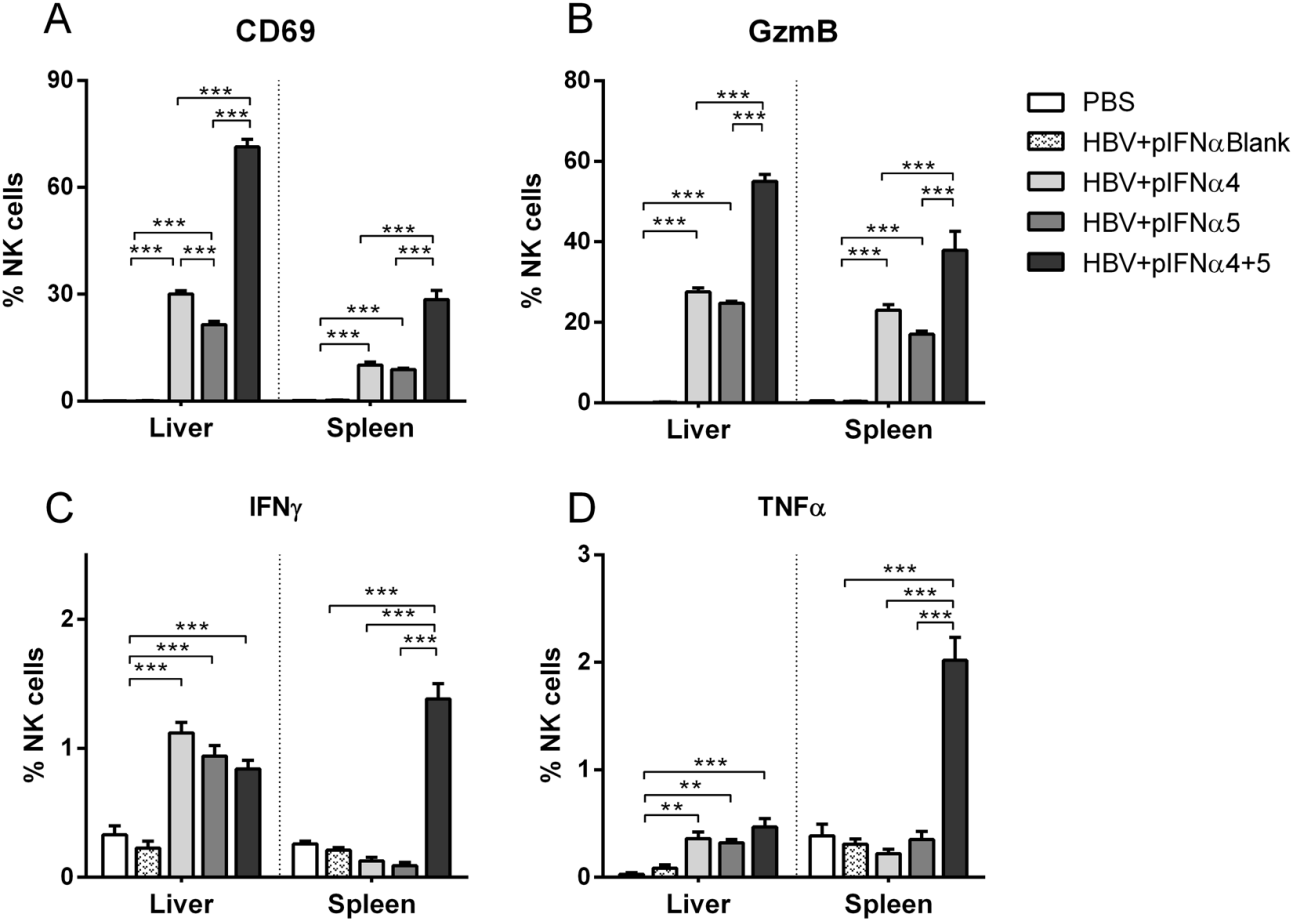
Analysis of NK cells from pIFNα4 or pIFNα5-treated mice. Mice received HI with 10 µg of pAAV-HBV1.2 plasmids in combination with 20 µg of plasmids encoding for IFNα subtypes (pIFNα4, pIFNα5, pIFNα4 + 5 or pIFNαB (empty vector)). Control mice received HI with PBS (PBS). At day 4 post HI mice were sacrificed and nucleated cells from liver and spleen were analyzed by flow cytometry for expression of NK cell-specific surface markers and intracellular cytokines. (**A**) The percentage of activated CD69^+^ NK cells, (**B**) GzmB, (**C**) IFNγ and (**D**) TNFα-producing NK cells are shown. At least six mice per group were analyzed. Differences between the groups were analyzed by using the One-way ANOVA, Statistically significant differences between the groups are indicated by ** for p < 0.01 and *** for p < 0.001.

Next, we determined the impact of pIFNα delivered by HI on NK cell responses in liver and spleen during HBV infection. At day 4 post HI, when IFNα was still detectable in the serum of pIFNα-treated mice (Fig. 5A), mice were sacrificed and liver and spleen cells were analyzed for NK cell activation and their effector phenotype. Endogenous expression of IFNα4 or IFNα5 strongly increased the frequencies of activated (Fig. 7A) and granzyme B (Fig. 7B) expressing NK cells in liver and spleen compared to control mice (HBV + pIFNαBlank). However, the combination of both IFNα-expressing plasmids additionally boosted NK cell responses and resulted in up to 71% activated CD69^+^ NK cells compared to 30% or 21% activated NK cells in pIFNα4 or pIFNα5 treated mice, respectively. Compared to IFN protein therapy (Fig. 3A and B), the endogenous expression of IFNα4 or IFNα5 remarkably improved NK cell responses in liver and spleen. The frequencies of IFNγ^+^ and TNFα^+^ NK cells were also significantly increased upon pIFNα injection (Fig. 7C and D). Taken together, NK cell responses are strongly induced by intrahepatic expression of either IFNα4 or IFNα5, however this effect was even more pronounced, if both IFNα subtypes are simultaneously expressed.

### T cell response was strongly induced by pIFNα

**Figure 8:**
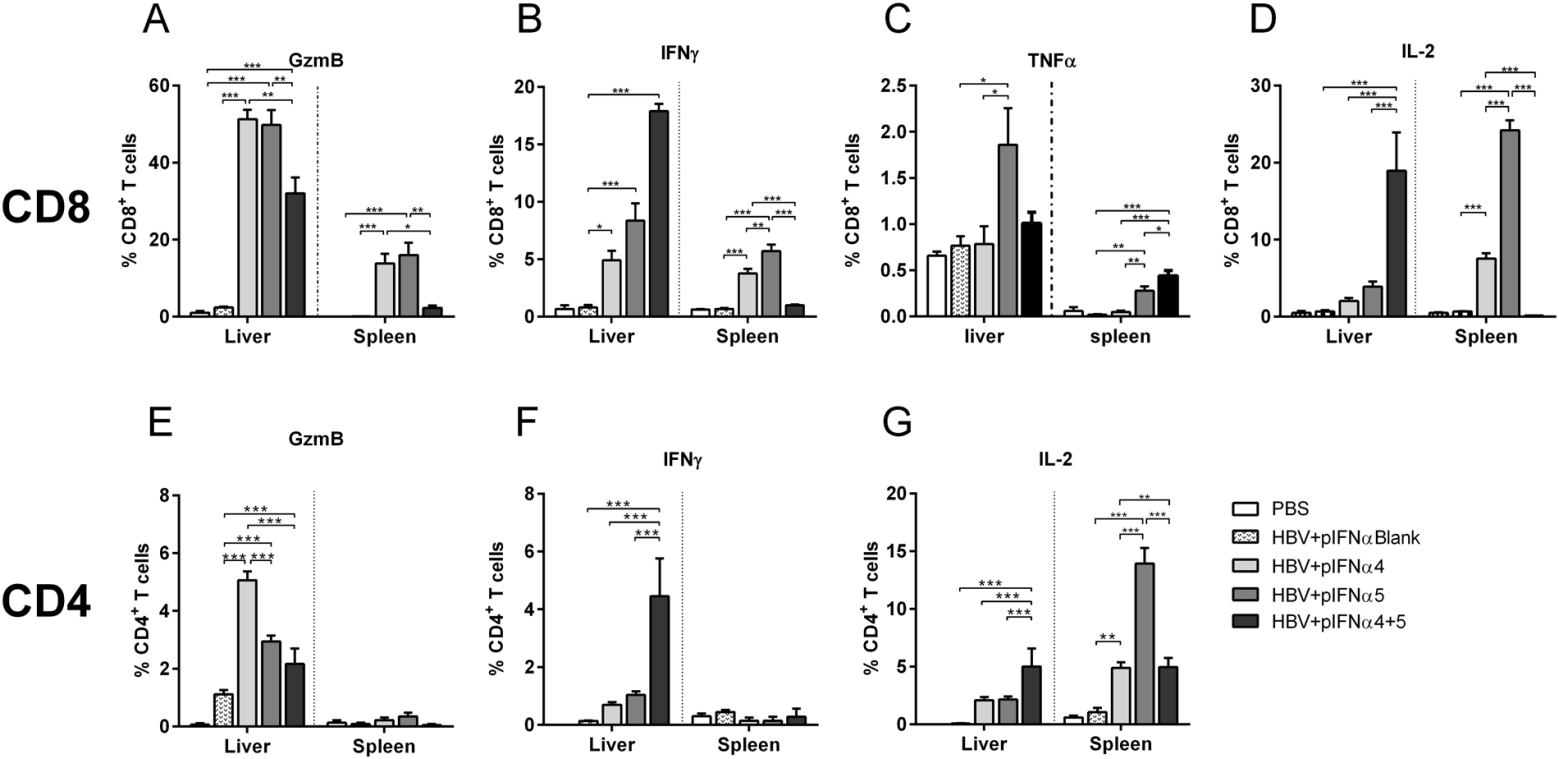
Analysis of CD8^+^ and CD4^+^ T cells from pIFNα4 or pIFNα5-treated mice. Mice received HI with 10 µg of pAAV-HBV1.2 plasmids in combination with 20 µg of plasmids encoding for IFNα subtypes (pIFNα4, pIFNα5, pIFNα4 + 5 or pIFNαBlank (empty vector)). Control mice received HI with PBS (PBS). At day 10 post HI mice were sacrificed and nucleated cells from liver and spleen were analyzed by flow cytometry for expression of T cell specific surface markers and intracellular cytokines. Frequencies of GzmB (**A**), IFNγ (**B**), TNFα (**C**) and IL-2-producing CD8^+^ T cells (**D**); GzmB (**E**), IFNγ (**F**) and IL-2-producing CD4^+^ T cells (**G**) are shown. At least four mice per group were analyzed. Differences between the groups were analyzed by using the One-way ANOVA. Statistically significant differences between the groups are indicated by * for p < 0.05, ** for p < 0.01 and *** for p < 0.001.

Finally, we determined the immunostimulatory potency of the endogenous expressed IFNα4 or IFNα5 during HBV infection *in vivo*. Therefore, at day 10 post HI, liver and spleen cells were analyzed for CD4^+^ and CD8^+^ T cell responses during HBV infection and pIFNα therapy. We also measured HBV-specific T cell responses in the spleen by ELIspot, but we did not detect any significant differences between the groups (data not shown). As only two HBV-specific peptides were used for ELIspot analysis, we further analyzed the whole population of activated effector T cells during acute HBV infection which might not be covered by these two epitopes. We stimulated splenocytes and liver cells *ex vivo* using αCD3/αCD28 stimulation to elucidate cytokine responses. Application of pIFNα4 or α5 significantly increased the frequencies of granzyme B expressing CD8^+^ T cells in the liver (Fig. 8A), whereas the combination of both plasmids did not further improve this response. In contrast, the IFNγ and IL-2 production of CD8^+^ T cells was particularly enhanced, if both plasmids were administered in parallel (Fig. 8B,D). The frequencies of TNFα producing CD8^+^ T cells were only marginally augmented by injection of pIFNα5 (Fig. 8C). Compared to IFNα protein treatment (Fig. 4A–D), the effector phenotype of CD8^+^ T cells was strongly improved upon pIFNα application. Furthermore CD8^+^ T cell responses were also detectable in the spleen, whereas daily treatment with recombinant IFNα4 or IFNα5 protein resulted in barely detectable T cell responses in the spleen of HBV HI mouse model.

CD4^+^ T cell responses were also significantly increased upon plasmid injections (Fig. 8E–G). The frequencies of granzyme B and IFN-γ producing CD4^+^ T cells were comparable to those measured in IFNα protein treated mice (Fig. 4E,F) and in both experimental setups, no splenic GzmB^+^ or IFNγ^+^ CD4^+^ T cells responses were detectable. In contrast, frequencies of IL-2 producing CD4^+^ T cells were strongly enhanced in the spleen of pIFNα treated mice (Fig. 8G), which was not seen in protein treated mice (Fig. 4G).

Our results clearly demonstrate, that different IFNα subtypes exhibit distinct antiviral activities against HBV *in vivo*. Daily treatment with IFNα4 or IFNα5 strongly increased ISG expression in the liver and improved NK and T cell responses. Changed experimental conditions (application of plasmids encoding for distinct IFNα subtypes) resulted in sustained intrahepatic IFNα expression which further improved ISG expression and host immune responses. In addition, combinational treatment with two different IFNα subtypes with distinct biological activities could further ameliorate host innate and adaptive immune responses in liver and spleen which led to pronounced reduction in HBV replication. Thus, detailed analyses of IFN-mediated immune responses are required to develop advanced immunotherapies which might better control and resolve HBV.

## Discussion

Little is known about the antiviral and immunomodulatory effects of different IFNα subtypes during HBV infection. In this study, we demonstrated that application of all IFNα subtypes except IFNα11 inhibited HBV replication *in vivo*, with IFNα4 and IFNα5 being the most effective subtypes. Decreased viral loads (Fig. 1) after therapeutic application of IFNα4 and IFNα5 correlated with induction of ISGs expression (Fig. 2) and expanded functional NK cells (Fig. 3) and T cells (Fig. 4) in both liver and spleen. Hydrodynamic injection of plasmids encoding for IFNα4 and IFNα5 was even more potent against HBV than injecting IFNα proteins. The combination of pIFNα4 and pIFNα5 showed a synergistic antiviral effect on HBV replication, with a strong increase in NK cell and T cell activity.

Numerous IFNα subtypes exist in all species and they all exhibit different effector functions. However the mechanism behind these differences remains elusive. Affinity to the receptor subunits and various downstream signaling events might partly explain the observed varying biological functions of the IFNα subtypes. Previous studies demonstrated that murine IFNα4 and IFNα9 had the highest antiviral potency against herpes simplex virus (HSV), while IFNα5 showed only a modest inhibitory effect on HSV replication^31^. During infection with murine cytomegalovirus (MCMV) application of plasmid encoding for IFNα6 reduced viral replication, while treatment with IFNα5 and IFNα2 expressing plasmids further increased MCMV infection *in vivo* ^32^. In addition, therapeutic treatment with IFNα11 significantly reduced viral loads during acute MCMV infection^33^. During acute Friend retrovirus infection in mice especially IFNα1 and IFNα11 significantly inhibited viral replication and improved NK and CD8^+^ T cell responses^33, 34^. Other studies in humanized mice infected with human immunodeficiency virus (HIV) clearly demonstrated that human IFNα14 strongly suppressed HIV replication, whereas the clinical relevant subtype IFNα2 was unable to reduce viral loads^35^. Our study clearly shows the different antiviral capacities of the various IFNα subtypes (Fig. 1) which is specific for HBV and completely differs from other viral infections.

One important mechanism by which IFNs mediate their antiviral effects is through the transcriptional regulation of relevant genes, such as ISGs^36^. IFNα induces several hundred ISGs, including ISG15, OAS and PRK, through the Jak/STAT signaling pathway^37–39^. In this study, HI of the HBV plasmid alone did not induce the expression of ISG15, OAS and PRK in the liver. This is consistent with the general concept that HBV is a stealth virus which does not directly activate the host’s innate immune system^40^. Moreover, it has been shown that HBV prevents induction of IFNα signaling and can interfere with transcription of ISGs in hepatocytes by inhibiting nuclear translocation of STAT1^41^. However, we could demonstrate that both IFNα4 and IFNα5 protein treatment and IFNα expression plasmids resulted in elevated ISG15, OAS and PRK expression in the liver of HBV HI mice, indicating the ability of these two subtypes of IFNα to overcome HBV-mediated impairment of IFNα signaling.

NK cells constitute 30–40% of intrahepatic lymphocytes and are crucial in defense against HBV infection^42^. Increasing numbers of circulating NK cells and higher cytotoxicity of hepatic NK cells were observed in acute HBV infection, suggesting their contribution to the initial viral suppression^15, 43^. In chronic HBV patients, altered phenotype and impaired function of NK cells were found^44, 45^. Although the cytotoxic capacity of NK cells is maintained, the IFNγ and TNFα production by NK cells, which play a major role in the early control of HBV replication, are strongly suppressed during chronic HBV infection^46^. Restoration of NK cell antiviral function by blocking immunosuppressive cytokines promotes HBV clearance^47^. NK cell activation and IFNγ production could also be partially restored by antiviral therapy through inhibition of viral replication^48^. In this study, we did not observe early NK activation in both liver and spleen in HBV HI mice. However, IFNα4 and IFNα5 treatment could strongly increase the activation, cytotoxic capacity and cytokine production of NK cells at early stage of HBV infection in these mice. This is consistent with previous observation in CHB patients that PegIFNα treatment was able to potently and cumulatively drive the proliferation and activation of NK cells^49^. Interestingly, co-application of pIFNα4 and pIFNα5 demonstrated a synergistic effect on inducing NK cell activation, which indicates that distinct mechanisms may be enrolled by these two IFNα subtypes to mediate NK cell activation during HBV infection.

It is general believed that CD8^+^ T cells are the major immune cells contributing to the clearance of HBV^50, 51^. Persistent HBV infection is associated with functional exhaustion of virus-specific CD8^+^ T cells^52^. This defect in virus-specific T cells is one of the primary reasons for the inability of the host to eliminate the persisting pathogen. Therefore, therapeutic strategies, which aim to enhance the patient’s own antiviral cellular immune response, have been considered as promising therapy^53^. However, current PegIFNα treatment showed no effect on modulating the HBV-specific T cell response in patients^54–56^. In contrast, we could demonstrate in HBV HI mouse model that IFNα4 and IFNα5 treatment led to a significant enhancement of cytotoxicity and cytokine production by T cells in both liver and spleen. Our results indicate that different IFNα subtypes may have diverse impact on modulating T cell response during HBV infection. Future examination of the effect of IFNα4 and IFNα5 treatment on improving antiviral T cell response in chronic HBV infection is needed. Notably, pIFNα4 and pIFNα5 HI treatment showed better effect on improving NK cell and T cell responses than IFNα4 and IFNα5 protein treatment. Compared to systemic IFNα treatment, pIFNα HI may result in higher intrahepatic IFNα concentration. Therefore, target delivering of IFNα to liver and maintaining a high intrahepatic IFNα concentration should be considered to optimize future IFNα treatment strategies in chronic hepatitis B patients.

## Methods

### HBV infectious clone and pIFNα expression plasmid

The plasmid pAAV-HBV1.2 containing 1.2-fold full length HBV genotype A genome was kindly provided by Prof. Pei-Jer Chen (National Taiwan University). The expression plasmid pkCMVint-mIFNα (pIFNα) contained the full length murine IFNα4 or IFNα5 gene as described before^57^. Large-scale plasmid preparations were obtained from cultures of transformed *Escherichia coli* (DH-5α) using NucleoBond® PC 2000 EF Kit (Macherey-Nagel, Dueren, Germany).

### Expression of IFNα subtypes and measurement of IFNα activity

HEK 293T cells grown in DMEM supplemented with 10% fetal bovine serum were transfected with each plasmid (pIFNα) by using the calcium phosphate method. At 3 days post transfection, supernatants were collected. To produce murine IFNα11, the stable cell line HEK293 IFNα11 was cultivated as described^58^. Protein expression was determined using an ELISA for mouse IFNα according to the manufacturer’s protocol (PBL Biomedical Laboratories, Piscataway, NJ, USA). In addition, murine IFNα subtype activity was determined by a virus-free, cell-based assay using Mx/Rage 7 cells as already described before^33, 58^. Cells were additionally stimulated with universal type I IFN (PBL Assay Science) as internal standard to calculate IFNα units.

### Preparation of HI mouse model

Adult BALB/C mice (male, 6–8 weeks old) were purchased from Harlan Laboratories, Germany, and maintained in a 12-hour light-dark cycle, and cared in accordance with national and local regulations. HI experiments were carried out as described previously^33, 59^. For IFNα protein-treated mice, 10 µg pAAV-HBV1.2 were injected into the tail vein of mice in a volume of 0.9% NaCl equivalent to 8% of the mouse body weight and the total volume was delivered within 5–8 s. Mice receiving pIFNα plasmids, were injected with 10 µg pAAV-HBV1.2 together with 20 µg pIFNα4, or 20 µg pIFNα5, or 10 µg pIFNα4 and 10 µg pIFNα5 by HI method. A plasmid without *IFNα* gene (pkCMVint backbone; pIFNαBlank) or PBS were used as control. Each experimental group included at least 12 mice. Experiments were performed either in China or in Germany and were were conducted in accordance with the Guide for the Care and Use of Laboratory Animals and were reviewed and approved by the local Animal Care and Use Committee (Animal Care Center, University of Duisburg-Essen, Essen, Germany, and Tongji Medical College, Huazhong University of Science and Technology, China).

### IFNα protein treatment *in vivo*

Mice were treated daily i.p. with 500 µl of medium containing 8000 units of IFNα1, α2, α4, α5, a6, α9, or α11 proteins daily from day −1 to 10 of HI. Control mice were injected with supernatant of HEK 293 T cells transfected with an empty vector pIFNαBlank (Medium). At 4, 10, 14 or 20 days post injection (dpi) the mice were sacrificed and analyzed for hepatic and splenic NK and T cell responses and antibody responses in the serum.

### Detection of IFNα proteins in mouse serum samples

Serum samples from the mice injected with IFNα plasmids were collected at 24 hours post injection (hpi), and at 4, 7 and 10 dpi. The amount of IFNα protein was determined by a commercial IFNα ELISA (PBL Biomedical Laboratories, Piscataway, NJ, USA) according to the manufacturer’s protocol. The IFNα standard and serum samples were measured in duplicates.

### Detection of HBsAg, HBeAg, HBV DNA HBsAb and HBcAb in mouse sera

The serum specimens were collected and assayed for HBsAg and HBeAg at 1, 4, 7 and 10 dpi. The levels of HBsAg, HBsAb, HBcAb and HBeAg in serum were detected by using either a commercial ELISA kit (Kehua, Shanghai, China) or an electrochemiluminescence immunoassay (ECLIA) on a modular analytics E170 analyzer (Roche, Indianapolis, IN, USA) according to the manufacturer’s instructions. 10-fold diluted serum samples were used for detection. Serum HBV DNA was extracted using a QIAamp MinElute Virus Spin kit (Qiagen, Hilden, Germany) and was quantitatively detected by real-time PCR (qPCR) using the SYBR green qPCR master mix (Qiagen, Hilden, Germany). Six mice were included per group. Melt curve analysis and agarose gel electrophoresis were used to verify the specificity of the qPCR. The following primers were used: forward primer: 5′-CTG CAT CCT GCT GCT ATG-3′ (nt 408–425), and reverse primer: 5′-CAC TGA ACA AAT GGC AC-3′ (nt 685–701) according to the reference sequence with GenBank accession number (AY220698.1). A serum sample containing a known concentration of HBV DNA was used as positive control.

### Immunohistochemistry

Liver tissue was taken from the mice at 4 and 10 dpi and embedded in paraffin. Intrahepatic HBcAg expression was visualized by immunohistochemical staining of tissue sections by polyclonal rabbit anti-HBcAg antibody (Dako, Glostrup, Denmark). The liver sections were also stained with hematoxylin. Staining was repeated three times for each sample.

### Purification of RNA from mouse liver tissue and Real-Time PCR detection

Total RNA was isolated from collected liver tissue at 4 dpi by tissue RNA extraction kit (OMEGA, Norcross, USA). RNA was reverse-transcribed and the product was used for analyzing the copy number of mouse ISG15, OAS, PKR mRNA by using Power SYBR® Green RNA-to-CT™ 1-Step Kit (Applied Biosystems). Primers for qPCR detection are provided by Qiagen Company (Qiagen, Hilden, Germany). β-actin was used as housekeeping gene to normalize qRT-PCR results.

### Cell isolation

Four or six mice per group were sacrificed at day 4 and 10 after HI. Preparation of single-cell suspensions of murine splenocytes was performed. Hepatic lymphocytes were isolated from the liver using published methods^60^ with some modifications. Briefly, livers were perfused with prewarmed PBS (to flush blood from the hepatic vasculature) and then forced through a 70 µm nylon cell strainer (BD Falcon, Franklin Lakes, NJ, USA). After washing with PBS, cell pellets were suspended in 5 ml of prewarmed enzyme solution, containing 0.05% Collagenase type II (Sigma-Aldrich, St. Louis, USA) and 500 U/ml DNase type I (Sigma-Aldrich, St. Louis, American) in Ca^2+^/Mg^2+^ free HBSS supplemented with 10% FBS, and digested for 40 min at 37 °C. Cells were then layered on 40% Percoll solution (Sigma-Aldrich, St. Louis, USA) in RPMI 1640 supplemented with 10 U/ml penicillin/streptomycin for density separation, and centrifuged at 300 × g for 17 minutes at 4 °C without brakes. Cell pellets were washed and suspended in 2 ml of Buffer EL (Qiagen, Hilden, Germany) to lyse red blood cells. Cell yields and viabilities were determined by trypan blue exclusion microscopy.

### *In vitro* stimulations of murine hepatic and splenic lymphocytes

To measure T cell activation and intracellular cytokine expression, 96-well microtiter plates were coated with 100 µl/well of 10 µg/ml mAb anti-CD3 (17A2, eBioscience, Hatfield, United Kingdom) overnight at 4 °C. Next day, the plate was washed twice and 2 × 10^6^ cells were stimulated in each well for 4.5 hours in the presence of 1 µg/ml of anti-CD28 antibody (clone 37.51, BD Pharmingen, Heidelberg, Germany) and 10 µg/ml of Brefeldin A (Sigma-Aldrich, St. Louis, USA). NK cells were stimulated with Ionomycin (500 ng/ml), PMA (25 ng/ml) and Brefeldin A (2 μg/ml) for 3 hrs at 37 °C.

### Cell surface and intracellular cytokine staining of murine splenic and hepatic lymphocytes

Cell surface staining of NK cells was performed using the anti-CD3 (clone 17A2, eBioscience, Hatfield, United Kingdom), anti-CD69 (clone H1.2F3, Invitrogen, Carlsbad, California, USA), anti-CD49b (clone DX5, BD Bioscience, Hatfield, United Kingdom) antibodies. Cell surface staining of T cells was performed using the anti-CD3 (clone 17A2, eBioscience, Hatfield, United Kingdom), anti-CD8 (100708, BioLegend) and anti-CD4 (clone GK1.5, BioLegend, San Diego, USA) antibodies. For intracellular cytokine staining cells were stimulated with either Ionomycin (500 ng/ml), PMA (25 ng/ml) and Brefeldin A (2 μg/ml) for NK cells or anti-CD3 (10 μg/ml), anti-CD28 (2 μg/ml) and Brefeldin A (2 μg/ml) for T cells for 4.5 h at 37 °C. After incubation, cells were fixed and permeabilized with CytoFix/CytoPerm (BD Bioscience) for 10 min. Intracellular cytokine stainings were performed with the following antibodies: anti-Granzyme B (clone GB11, BioLegend, San Diego, USA), anti-IFNγ (clone XMG1.2, BioLegend, San Diego, USA), anti-TNFα (clone MP6-XT22, BioLegend, San Diego, USA) and anti-IL-2 (clone JES6-5H4, BioLegend, San Diego, USA). Dead cells were excluded from analysis via fixable viability dye (eBioscience, Hatfield, United Kingdom). Data were acquired on LSR II flow cytometer (Becton Dickinson, Heidelberg, Germany) from 500,000 lymphocyte-gated events per sample. Analyses were performed using FlowJo software (Tree Star, Ashland, USA).

### Statistical analysis

Experimental data were reported as means ± standard deviations. Nonparametric one-way ANOVA was used with Dunn’s multiple comparisons (GraphPad Prism software; GraphPad, San Diego, CA) to compare different groups in our study.

## Electronic supplementary material

Supplementary figure 1
