# Supplementary material for: Different antiviral effects of IFNα subtypes in a mouse model of HBV infection
Source: Sci Rep. 2017 Mar 23;7:334. doi: 10.1038/s41598-017-00469-1 (PMC5428457; doi:10.1038/s41598-017-00469-1)
Supplement: Supplementary file 1 — Supplementary figure 1 [file 41598_2017_469_MOESM1_ESM.pdf]

Different antiviral effects of IFNα subtypes in a mouse model of HBV infection

Jingjiao Song[1,2], Sheng Li[2], Yun Zhou[2], Jia Liu[2], Sandra Francois[3], Mengji Lu[3], Dongliang Yang[2], Ulf Dittmer[3], Kathrin Sutter[3]

1. Experimental Medicine Center, Tongji Hospital, Tongji Medical College, Huazhong University of Science and Technology, Wuhan, P.R. China,
2. Department of Infectious Diseases, Union Hospital of Tongji Medical College, Huazhong University of Science and Technology, Wuhan, P.R. China,
3. Institute of Virology, University Hospital of Essen, University of Duisburg-Essen, Essen, Germany

Correspondence to Jingjiao Song (jjsong@tjh.tjmu.edu.cn)

Supplementary Information

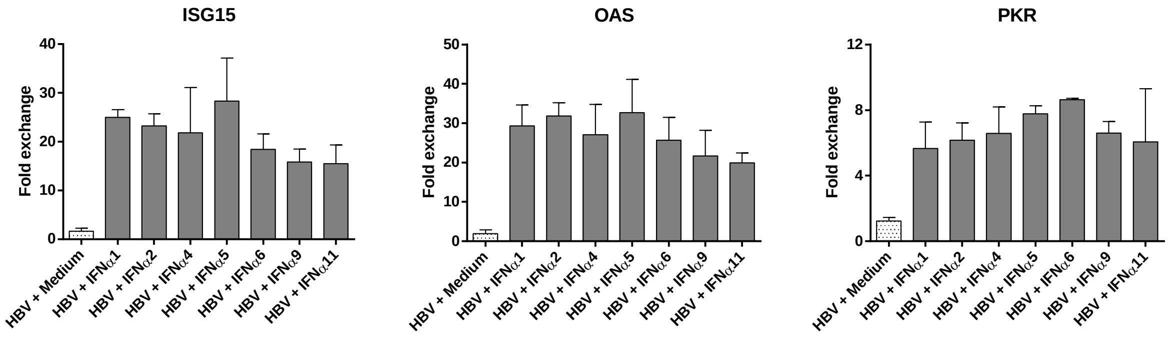

<u>Supp. Fig. 1: Induction of ISG15, OAS and PKR expression post treatment with different IFNα subtype proteins.</u>

Mice received HI with 10 µg of pAAV-HBV1.2 plasmids. Mice were treated i.p. with 8000 units of recombinant IFNα1, α2, α4, α5, α6, α9 or α11 starting from days -1 to 3 post HI. Control mice were treated i.p. with media (HBV+Medium). Total RNA was extracted from liver tissue at day 4 post HI and the mRNA expression levels of ISG15, OAS and PKR were determined by qRT-PCR. The β-actin mRNA expression was quantified for normalization. Each sample was run in duplicate and at least three mice per group were analyzed.
